# *Helicobacter hepaticus* Induces an Inflammatory Response in Primary Human Hepatocytes

**DOI:** 10.1371/journal.pone.0099713

**Published:** 2014-06-16

**Authors:** Moritz Kleine, Tim Worbs, Harald Schrem, Florian W. R. Vondran, Alexander Kaltenborn, Jürgen Klempnauer, Reinhold Förster, Christine Josenhans, Sebastian Suerbaum, Hüseyin Bektas

**Affiliations:** 1 Department of General, Visceral and Transplant Surgery, Hannover Medical School, Hannover, Germany; 2 Institute of Immunology, Hannover Medical School, Hannover, Germany; 3 Institute of Medical Microbiology and Hospital Epidemiology, Hannover Medical School, Hannover, Germany; 4 Federal Armed Forces Medical Centre, Hannover, Germany; National Institutes of Health, United States of America

## Abstract

*Helicobacter hepaticus* can lead to chronic hepatitis and hepatocellular carcinoma in certain strains of mice. Until now the pathogenic role of *Helicobacter species* on human liver tissue is still not clarified though *Helicobacter species* identification in human liver cancer was successful in case controlled studies. Therefore we established an *in vitro* model to investigate the interaction of primary human hepatocytes (PHH) with *Helicobacter hepaticus*. Successful co-culturing of PHH with *Helicobacter hepaticus* was confirmed by visualization of motile bacteria by two-photon-microscopy. Isolated human monocytes were stimulated with PHH conditioned media. Changes in mRNA expression of acute phase cytokines and proteins in PHH and stimulated monocytes were determined by Real-time PCR. Furthermore, cytokines and proteins were analyzed in PHH culture supernatants by ELISA. Co-cultivation with *Helicobacter hepaticus* induced mRNA expression of Interleukin-1 beta (IL-1β), Tumor necrosis factor-alpha, Interleukin-8 (IL-8) and Monocyte chemotactic protein-1 (MCP-1) in PHH (p<0.05) resulting in a corresponding increase of IL-8 and MCP-1 concentrations in PHH supernatants (p<0.05). IL-8 and IL-1β mRNA expression was induced in monocytes stimulated with *Helicobacter hepaticus* infected PHH conditioned media (p<0.05). An increase of Cyclooxygenase-2 mRNA expression was observed, with a concomitant increase of prostaglandin E2 concentration in PHH supernatants at 24 and 48 h (p<0.05). In contrast, at day 7 of co-culture, no persistent elevation of cytokine mRNA could be detected. High expression of intercellular adhesion molecule-1 on PHH cell membranes after co-culture was shown by two-photon-microscopy and confirmed by flow-cytomety. Finally, expression of Cytochrome P450 3A4 and albumin mRNA were downregulated, indicating an impairment of hepatocyte synthesis function by *Helicobacter hepaticus* presence. This is the first *in vitro* model demonstrating a pathogenic effect of a *Helicobacter spp*. on human liver cells, resulting in an inflammatory response with increased synthesis of inflammatory mediators and consecutive monocyte activation.

## Introduction

*Helicobacter hepaticus* (*H. hepaticus*), a member of the enterohepatic group of *Helicobacter* species, can cause hepatitis and consecutive hepatic dysplasia and hepatocellular carcinoma (HCC) in several strains of mice [1]. Experimental infection of A/JCr mice revealed colonisation of the upper gastrointestinal tract followed by infiltration of the bile duct and the liver [2] resulting in a multifocal necrosis of hepatocytes with infiltration of lymphocytes, macrophages and neutrophils, especially around the bile ducts. *H. hepaticus* was predominantly seen in bile canaliculi resulting in Kupffer cell hyperplasia and proliferation of bile ducts [3]. *H. hepaticus* has not been detected in humans. However, nine of ten case controlled studies have linked the identification of Helicobacter-specific DNA in the liver to the development of HCC [4]. Further, liver diseases in which *Helicobacter* species (spp.) DNA has been detected include primary sclerosing cholangitis, and primary biliary cirrhosis [5]. Although the role of *Helicobacter spp*. in the pathogenesis of these diseases remains unclear, available data support the notion that *Helicobacter* co-infection may play a role in hepatic carcinogenesis associated with hepatitis viruses [6]. The strength of the association between *Helicobacter* spp. and the development of liver malignancy increases with the severity of hepatopathy [6]. *In vivo* experiments with mice infected with *H. hepaticus* wild-type versus cytolethal distending toxin mutants revealed a toxin dependent promotion from inflammation to dysplasia. The transcription of proinflammatory markers (Tumor necrosis factor-alpha (TNFα), Interferone-gamma and Cyclooxygenase-2 (COX-2)) was significantly induced in both groups as compared to controls with higher cytokine levels in wild-type *H. hepaticus* infected animals [7].

Hepatic inflammation is a finding in many liver injuries leading to acute or chronic hepatitis. Liver injury can for example be induced by viral hepatitis, endotoxins, alcoholic or ischemia-reperfusion. The release of pro-inflammatory cytokines from resident macrophages (Kupffer cells) and hepatocytes has been shown to represent an important pathogenic factor in inflammatory liver disease [8]. Inflammatory cytokines (TNFα, IL-1 and IL-8) not only contribute to the early phase of the disease but as well cause sustained liver inflammation [9]. The resulting activation of immune cells (e.g. monocytes, neutrophils and lymphocytes) is paramount to the development of liver injury, and neutrophil and monocyte accumulation in the liver is triggered by TNFα/IL-1 and MCP-1, respectively [10]–[11]. How certain agents, such as hepatotropic bacteria, trigger a specific cytokine response as well as the underlying kinetics of the inflammatory cytokine network during an ensuring immune reaction in the liver are still only partially understood. Especially the pathogenic effects of *Helicobacter spp*. on human liver cells are still completely unclear.

In this context, the present study investigates the influence of *H. hepaticus* on primary human hepatocytes and the resulting inflammatory response.

## Results

### Two-photon-microscopy of successful co-cultivation of *H. hepaticus* with PHH

**Figure 1:**
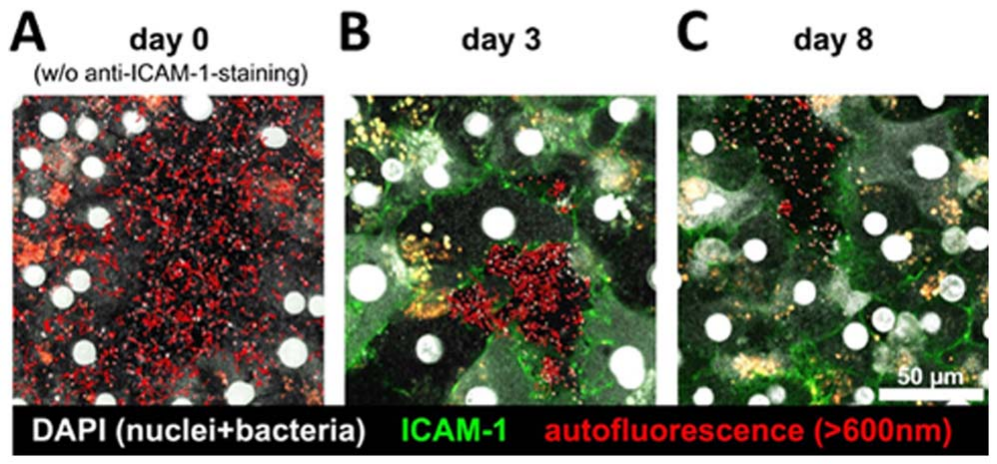
Two-photon-microscopy of *H. hepaticus* ATCC 51449 co-cultivation with primary human hepatocytes (PHH). PHH were co-cultured with *H. hepaticus* at a MOI of 100 per hepatocyte for different time intervals (6 h (A), 3 d (B), or 8 d (C)). ICAM-1 on hepatocyte cell membranes was stained using an AlexaFluor488-labeled anti-human-ICAM-1-antibody (green) on cultures of day 3 and 8. Cell nuclei and *H. hepaticus* were stained with DAPI (white). Starting positions of individual bacteria are marked with red dots, tracks of bacterial movement are displayed as red lines (see also Video S1–S3). (A) At 6 hours of co-cultivation (day 0), numerous bacteria are present in the vicinity of hepatocytes, displaying a highly active, seemingly random motility (see also Video S1). (B) Following 3 days of co-cultivation (day 3), high levels of ICAM-1 are expressed on hepatocyte cell membranes, while most bacteria present in the culture surrounding PHH continue to exihibit a high overall motility (see also Video S2). (C) After 8 days of infection (day 8), ICAM-1-staining on hepatocytes is comparable to day 3, while the remaining bacteria were observed to be largely non-motile (see also Video S3). Snapshots of representative view fields of the co-culture setup. 2-PM settings: TiSa laser at 800 nm, 485 and 600 nm long pass filters, imaging volume 150×150 µm (XY), 12–40 µm, 2–4 Z-slices (Z), acquisition speed 1 frame/3–6 sec, 40× replay speed.

Over a cultivation period of 8 d, PHH displayed a polygonal, differentiated cell morphology. PHH cell membranes remained intact, and there was no obvious difference in hepatocyte morphology between control cultures and hepatocytes co-cultured with *H. hepaticus* during the time period under investigation. The seemingly random spontaneous motility of *H. hepaticus*, which can be seen as a surrogate marker for bacterial viability and fitness, was highest after 6 h of co-cultivation (Fig. 1A, Video S1), however, also after 3 d many bacteria still displayed a high degree of motility (Fig. 1B, Video S2). After 8 d, the number of intact *H. hepaticus* present in the co-culture setup was clearly reduced, and most, but not all, of the bacteria present were found to be largely immotile (Fig. 1C, Video S3), indicating a strongly diminished bacterial viability.

### PHH express acute phase cytokines as a response to co-cultivation with *H. hepaticus*

**Figure 2:**
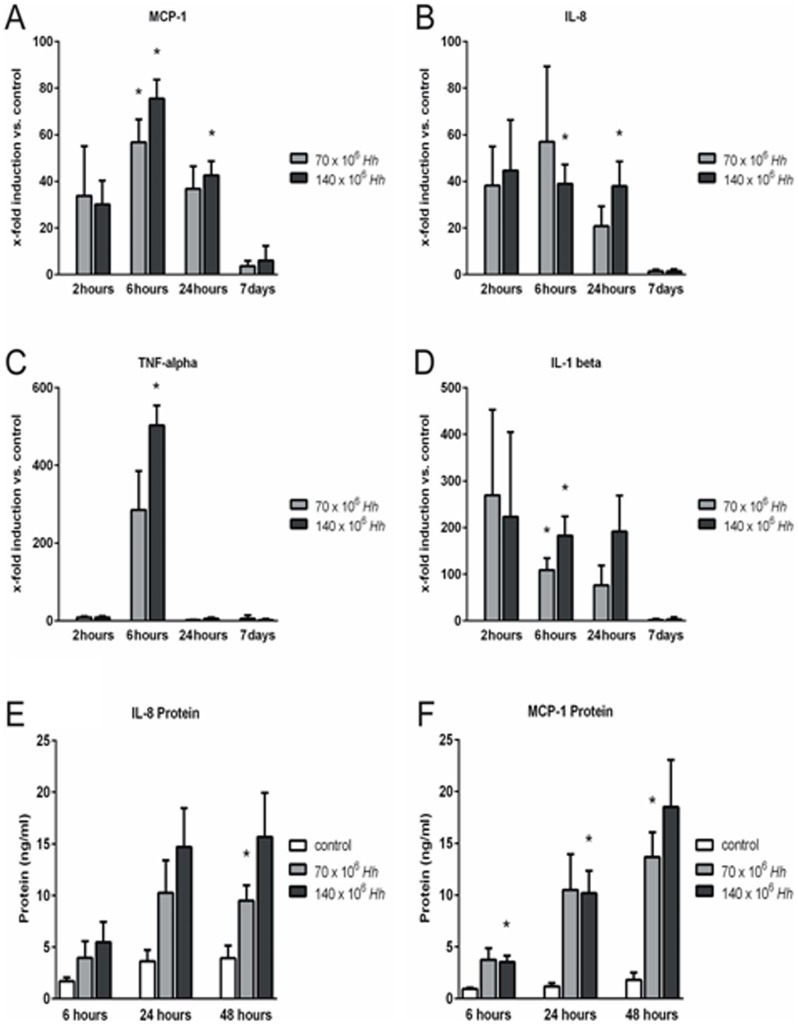
Primary human hepatocytes (PHH) express acute phase cytokines in response to co-cultivation with *H. hepaticus*. PHH were co-cultured with *H. hepaticus ATCC 51449* at a MOI of 50 (70×10^6^ Hh) or 100 (140×10^6^ Hh) bacteria per hepatocyte (n = 3). Real-time RT PCR of hepatocytes revealed increased mRNA expression of the acute phase cytokines Monocyte chemotactic protein 1 (MCP-1) (A), Interleukin 8 (IL-8) (B), Tumor necrosis factor alpha (TNF-alpha) (C) and Interleukin 1 beta (IL-1 beta) (D). Cytokine mRNA is expressed as the fold induction compared to untreated control culture from the same hepatocyte donor for each time point (A-D). Up-regulation of acute phase cytokine mRNA in hepatocytes resulted in an increased IL-8 (E) and MCP-1 (F) concentration in the supernatant of infected PHH as measured by ELISA. Data shown as mean±SEM, n = 3. * = p<0.05 versus control.

The strongest increase in acute phase cytokine expression was found after 6 and 24 h of co-cultivation with *H. hepaticus* at a high MOI of 100 bacteria per hepatocyte for MCP-1 (p = 0.012 and p = 0.020, respectively) and IL-8 (p = 0.010 and p = 0.025, respectively), and after 6 h for TNFα (p = 0.010) and IL-1β (p = 0.048) (Fig. 2A-D). In this context, it is remarkable that TNFα was the only cytokine with a pronounced transient increase of mRNA levels at 6 h of co-cultivation, in contrast to MCP-1, IL-8 and IL-1β for which a persistent increase in mRNA expression beyond 24 h was observed. For all acute phase cytokines analysed, the increase of mRNA expression was abolished completely after 7 d of co-cultivation (Fig. 2A–D).

IL-8 protein concentrations in PHH supernatants (MOI, 50 or 100 bacteria per hepatocyte) increased gradually over time, showing a substantial difference compared to untreated controls at 24 and 48 h (Fig. 2E). Increases in MCP-1 protein concentrations in PHH supernatants at 6, 24 and 48 h of co-cultivation correlated well with the respective mRNA expression levels at earlier time points. Peak levels of MCP-1 mRNA expression were reached at 6 h of co-cultivation, then decreasing over time. In contrast, MCP-1 protein concentrations in the supernatant of co-cultured PHH were persistently increased beyond 48 h (p = 0.040, p = 0.041 and p = 0.029, respectively, Fig. 2F).

### *H. hepaticus* induces COX-2 expression, resulting in an increased PGE2 synthesis by PHH

**Figure 3:**
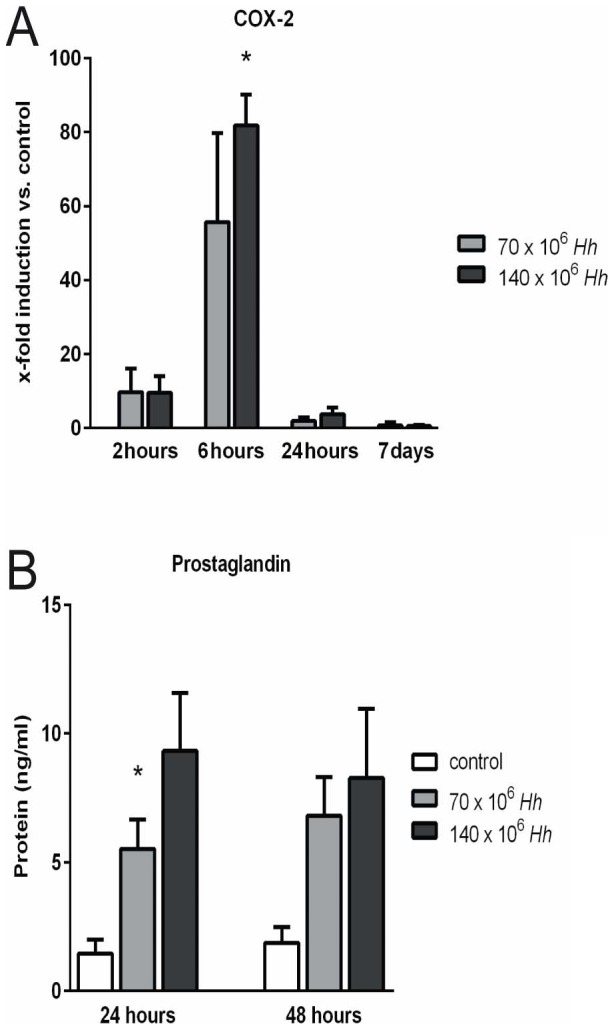
*H. hepaticus* ATCC 51449 induces an early transient COX-2 expression, resulting in an increased PGE2-synthesis by primary human hepatocytes (PHH). PHH were co-cultured with *H. hepaticus* at a MOI of 50 (70×10^6^ Hh) or 100 (140×10^6^ Hh) bacteria per hepatocyte (n = 3). (A) Real-time RT PCR of hepatocytes revealed significantly increased mRNA expression of COX-2 6 hours after infection. COX-2 mRNA is expressed as the fold induction compared to untreated control culture from the same hepatocyte donor for each time point. (B) Induction of COX-2 led to an increased synthesis of PGE2 by PHH as measured in the supernatant of co-cultured hepatocytes by ELISA with maximum concentration at 24 hours after infection with *H. hepaticus* at a MOI of 100 cells per hepatocyte (n = 3). * = p<0.05 versus control.

Real-Time PCR revealed a pronounced peak of COX-2 mRNA expression at 6 h of PHH co-cultivation with *H. hepaticus*. Interestingly, this effect was very short-lived, as only 18 h later, COX-2 mRNA levels had nearly returned to baseline, and on day 7 of co-culture, no difference between treatment and control groups was detectable (Fig. 3A). PGE2, end product of the enzyme COX-2, was increased in the PHH supernatant at 24 and 48 h of co-cultivation depending on the MOI of *H. hepaticus* (50 versus 100 bacteria per hepatocyte) (Fig. 3B).

### ICAM-1 mRNA expression and ICAM-1 incorporation into PHH cell membranes are increased after *H. hepaticus* exposure

**Figure 4:**
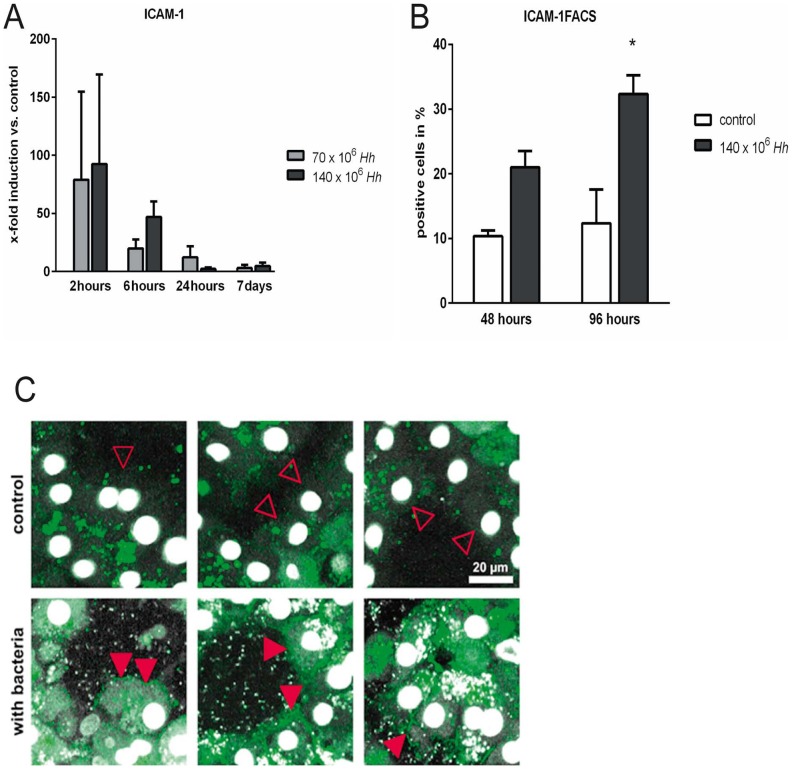
ICAM-1 mRNA expression and ICAM-1 incorporation into primary human hepatocytes (PHH) cell membranes are increased after *H. hepaticus* exposure. PHH were co-cultured with *H. hepaticus* at a MOI of 50 (70×10^6^ Hh) or 100 (140×10^6^ Hh) bacteria per hepatocyte (n = 3). (A) Real-time RT PCR of hepatocytes showed increased mRNA expression of ICAM-1 2 and 6 hours after infection. ICAM-1 mRNA expression is displayed as x-fold induction compared to untreated control cultures from the same hepatocyte donor for each time point. (B) PHH were co-cultured with *H. hepaticus* for 48 and 96 hours at a MOI of 100 per hepatocyte. Subsequently, hepatocytes were harvested, stained with an AlexaFluor488-labeled anti-human ICAM-1 antibody, and analyzed by flow cytometry. Flow cytometry results confirmed an increased incorporation of ICAM-1 into cell membranes of PHH under co-culture conditions with *H. hepaticus*. * = p<0.05 versus the control culture. (C) After 48 hours of PHH culture in the absence or presence of *H. hepaticus* at a MOI of 100 per hepatocyte, cell surface staining for ICAM-1 was performed with an AlexaFluor488-labeled anti-human ICAM-1 antibody (green). *H. hepaticus* and hepatocyte nuclei were stained with DAPI (white). Representative snapshots are shown taken from two-photon microscopy time lapse recordings of PHH cultures in the absence (control, upper row) or presence (with bacteria, lower row) of *H. hepaticus* (n = 3). Aquisition settings (TiSa laser tuned to 760 nm, 485 nm long pass filter, no bandpass filters) and snapshot image processing were identical for all recordings. While surface ICAM-1 staining was virtually absent in control cultures (open red arrow heads), PHH cultivated in the presence of *H. hepaticus* displayed a marked ICAM-1 expression on the cell surface (filled red arrow heads).

*H. hepaticus* induced a very early (2 h) increase of ICAM-1 mRNA expression in PHH, although the degree of mRNA induction was very heterogeneous in between hepatocyte donors. Over time, the induction effect decreased, and at day 7, ICAM-1 mRNA expression was nearly back to control levels (Fig. 4A). To further investigate whether this early increase of mRNA expression correlated with protein synthesis and ICAM-1 incorporation into PHH cell membranes, we used AlexaFluor488-labeled anti-human-ICAM-1-antibodies to stain the adhesion molecule on cell surfaces. Flow cytometry revealed a time dependent increase in ICAM-1 surface expression under co-culture conditions as compared to control cultures (Fig. 4B, p = 0.049). This increase in ICAM-1 surface levels appeared delayed as compared to peak ICAM-1 mRNA expression levels in PHH. Furthermore, we used 2-PM to visualize PHH after 48 h of *in vitro* culture in the absence or presence of *H. hepaticus*, finding a pronounced surface expression of ICAM-1 after incubation with *H. hepaticus* (Fig. 4C).

### *H. hepaticus* impairs physiological cell functions of PHH

**Figure 5:**
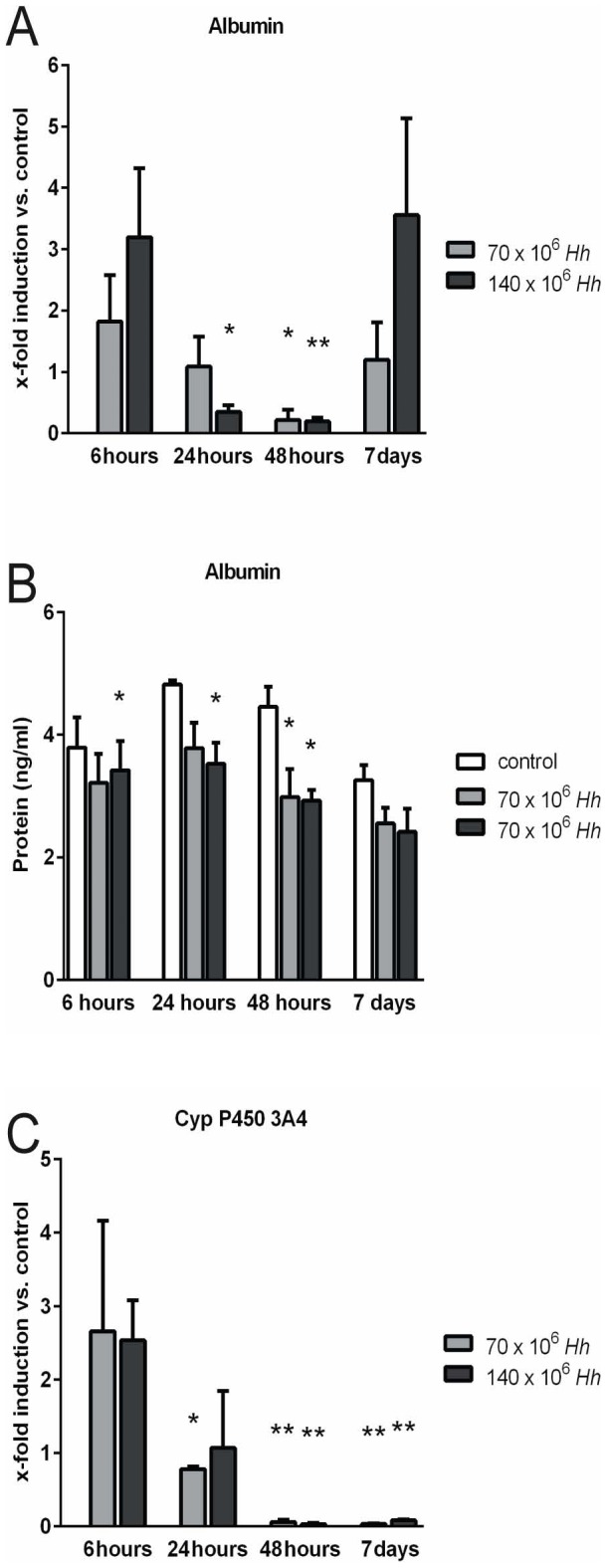
*H. hepaticus* impairs physiological cell functions of primary human hepatocytes (PHH). PHH were co-cultured with *H. hepaticus* at a MOI of 50 (70×106 Hh) or 100 (140×106 Hh) bacteria per hepatocyte (n = 3). (A) Albumin mRNA expression in PHH decreased after an initial, transient increase, with lowest levels at 48 hours of co-cultivation with *H. hepaticus* at a MOI of 100 bacteria per hepatocyte returning to unchanged or even higher levels on day 7. (B) The downregulation in albumin gene expression in PHH resulted in a slight, but statistically significant reduction of albumin concentration in the supernatant of co-cultured hepatocytes 6, 24 and 48 hours after infection as measured by ELISA. (C) Real-time RT PCR of hepatocytes revealed significantly decreased mRNA expression of cytochrome P450 3A4 24, 48 hours and 7 days after infection. Albumin and CYP 3A4 mRNA is expressed as the fold induction compared to untreated control culture from the same hepatocyte donor for each time point. (n = 3) * = p<0.05 and ** = p<0.005 versus control.

After an initial transient increase, albumin mRNA expression in PHH decreased over time with lowest levels at 48 h of co-cultivation with *H. hepaticus* at a MOI of 100 bacteria per hepatocyte (p = 0.026 and p = 0.005, respectively). On day 7, albumin mRNA expression returned to comparable or higher levels in co-cultured PHH as compared to control cultures (Fig. 5A). Measurement of albumin in cell culture supernatants confirmed these findings at the protein level, with lower albumin concentrations under co-culture conditions at 6, 24 and 48 h (p = 0.046, p = 0.046 and p = 0.013, respectively) (Fig. 5B).

The most significant reduction of CYP3A4 mRNA expression in PHH following infection with *H. hepaticus* at a MOI of 50 and 100 bacteria per hepatocyte was detected after 48 h (p = 0.001 and p<0.001, respectively) and 7 d (p<0.001 and p<0.001, respectively). During follow up of 7 d, mRNA expression of CYP3A4 did not recover to control levels (Fig. 5C).

### No impact of *H. hepaticus* on AST release from PHH

AST levels in the supernatant of co-cultivated and control PHH increased continuously, reaching a steady state after 24 h of co-cultivation, without further changes until day 7. Apart from slightly higher – though not statistically significant – AST levels at 30 min and 2 h of co-cultivation, no difference between infection and control group was observed at any time point of culture (data not shown). Thus, a major direct cytotoxic effect on PHH due to *H. hepaticus* infection can largely be excluded.

### *H. hepaticus* infected PHH conditioned media activate monocytes

**Figure 6:**
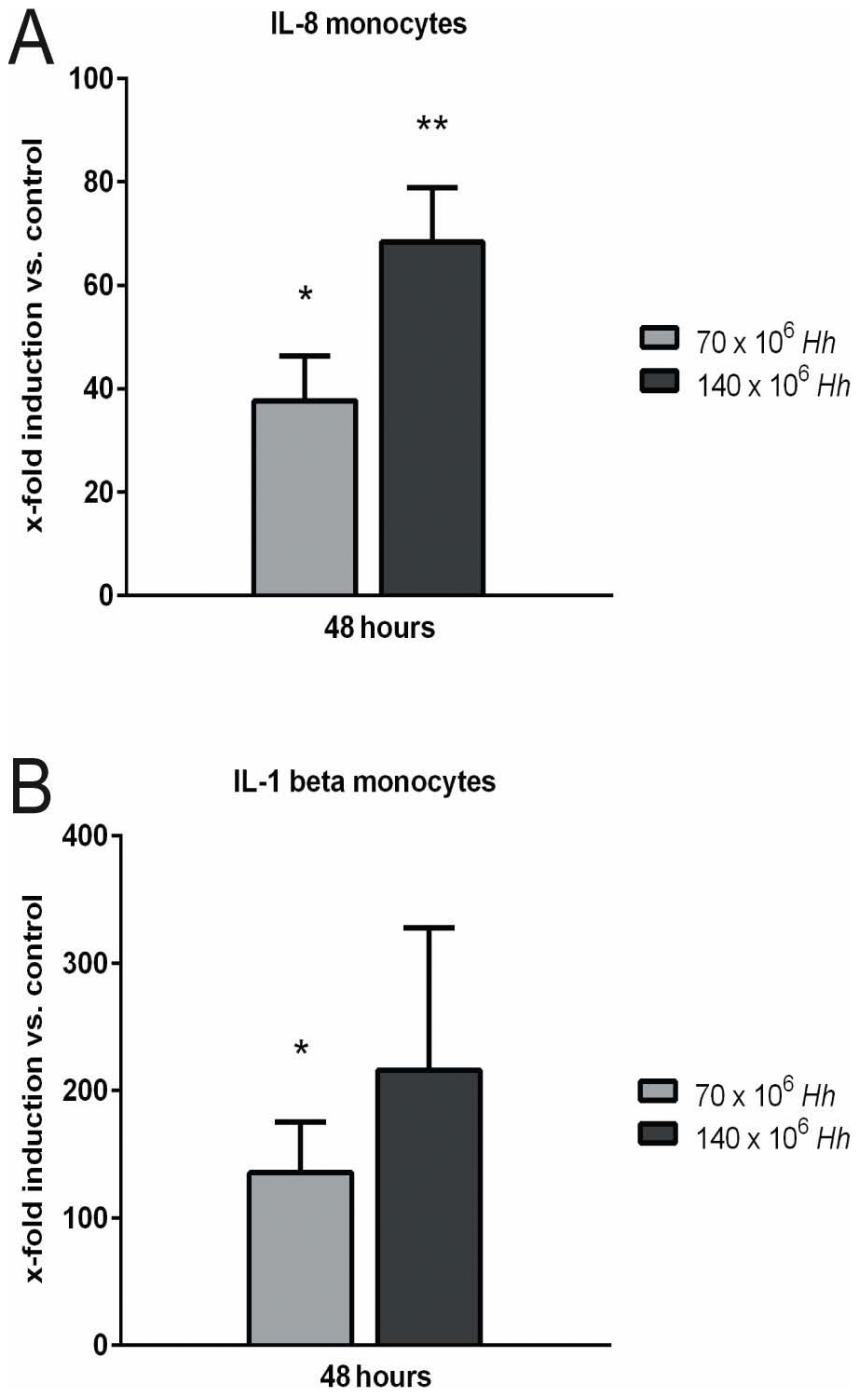
Primary human hepatocyte (PHH) conditioned media infected with *H. hepaticus* activate monocytes. Human CD14^+^ monocytes were isolated from peripheral blood of healthy donors and seeded at 10.000 cells/well. Monocytes were stimulated 24 hours with 100 µl of centrifuged PHH conditioned media infected with *H. hepaticus* at a MOI of 50 (70×10^6^ Hh) or 100 (140×10^6^ Hh) bacteria per hepatocyte for 48 hours (n = 3). Real-time RT PCR of monocytes revealed a dose-dependent increase of the mRNA expression of (A) Interleukin-1 beta (IL-1 beta) and (B) Interleukin-8 (IL-8). Cytokine mRNA is expressed as the fold induction compared to untreated control culture from the same hepatocyte donor for each time point (A-B). (n = 3). * = p<0.05 and ** = p<0.005 versus control.

We found a significant up-regulation of IL-8 mRNA in monocytes after treatment with PHH conditioned media following infection with *H. hepaticus* at a MOI of 50 or 100 bacteria per hepatocyte for 48 h (p = 0.025, p = 0.008, respectively) (Fig. 6A). Furthermore, IL-1β mRNA was also up-regulated in monocytes following the same treatment (p = 0.044, p = 0.150, respectively) (Fig. 6B).

## Discussion

Establishing a new *in vitro* model of liver inflammation, this study characterizes for the first time the acute phase reaction of PHH cytokine expression and release induced by co-cultivation with *H. hepaticus*. IL-1β, TNFα, MCP-1 and IL-8 mRNA are increasingly expressed in hepatocytes as early as 6 h after infection with *H. hepaticus*. These changes in mRNA expression correlate well with increased concentrations of MCP-1 and IL-8 in the co-culture supernatant 24 h after bacterial infection. Interestingly, mRNA expression kinetics differed between cytokines, with a short transient induction of TNFα returning to control levels already at 24 h. In contrast, IL-1β, MCP-1 and IL-8 mRNA induction persisted for more than 24 h (Fig. 2A-F). This pattern of early acute phase cytokine expression and excretion by hepatocytes is in line with earlier reports on inflammatory cytokine expression during various inflammatory liver injuries [12]–[13]. Functionally, it is well established that cytokines such as TNFα and IL-1 represent important triggers for neutrophil accumulation within liver sinusoids [14], and neutrophil transmigration into the space of Dissé [15]. The chemotactic signal of IL-8 was shown to be a potent chemoattractant for neutrophils and plays a major role in acute hepatic inflammation and chronic liver disease [16], and MCP-1 regulates the recruitment of monocytes into liver tissue [10]. Importantly, following co-cultivation of PHH with *H. hepaticus*, we found a rapid transient increase in mRNA and cytokine concentrations of all these inflammatory cytokines.

Treatment of PHH with *H. hepaticus* at a MOI of 50 versus 100 bacteria resulted in an only minor further increase in cytokine mRNA and protein induction, which might indicate that the signaling pathways involved were already saturated after treatment of PHH at the lower MOI of *H. hepaticus* (Fig. 2A-D). As a control, we performed co-culture experiments with even lower MOIs of 1 and 10 bacteria per hepatocyte, respectively, and indeed found a strong correlation between the amount of applied bacteria and the level of inflammatory cytokine expression in infected PHH (data not shown). We conclude that hepatocytes react quite sensitive towards bacteria/bacterial Pathogen-associated molecular patterns (PAMP), and are probably able to initiate inflammatory cytokine responses at much lower “bacterial burdens” in the in vivo situation. In this context, further studies on hepatocyte Toll-Like-Receptors and downstream proteins of cellular signaling in our newly established PHH infection model could provide insights into the specific signaling molecules activated in PHH due to PAMP recognition following *H. hepaticus* treatment.

Our data show that infection of PHH with *H. hepaticus* leads to the up-regulation of ICAM-1 as shown by Real-Time PCR, 2-PM and FACS (Fig. 4A-C). Probably due to the very heterogeneous induction of ICAM-1-mRNA in PHH from different human donors, the rapid initial increase after only 2 h of co-culture with *H. hepaticus* was not statistically significant (Fig. 4A). Nonetheless, 2-PM and FACS analysis clearly indicate an increase of ICAM-1 cell surface expression on PHH after treatment with *H. hepaticus* (Fig. 4B+C, Videos S2 and S3). *In vitro*, neutrophils have been shown to use β2-integrins to establish contacts with cytokine-stimulated hepatocytes via ICAM-1 [17]. It is assumed that these β2-integrin-dependent contacts as well trigger the release of reactive oxygen species, resulting in cell death [18]. Earlier data from a murine model suggested a TNFα- and IL-1-mediated expression of ICAM-1 on hepatocytes following *Salmonella* infection [19]. In contrast, our cell culture model of PHH revealed that ICAM-1 mRNA expression in hepatocytes is increased already 2 h after infection with *H. hepaticu*s, without additional stimulation of acute phase cytokines by bystanding immunological cells (Fig. 4A). We thus conclude that *H. hepaticus* is able to directly induce the expression of acute phase cytokines and ICAM-1 in infected hepatocytes via cytokine-independent mechanisms. In this scenario, direct regulation of hepatocyte gene expression, for example via toll-like receptors expressed on PHH, might represent the mechanism of action by which inflammation is initiated [20], and should therefore be the aim of future studies.

Based on our finding that *H. hepaticus* can induce a very early expression of pro-inflammatory mediators in PHH we investigated their impact on monocyte activation. It is known that hepatic macrophages (Kupffer cells) contribute to acetaminophen (APAP)-induced hepatotoxicity through the production of inflammatory cytokines e.g. TNFα and IL-1β [21]. In addition to these well described resident hepatic macrophages, a second population of migratory macrophages was described that infiltrates the liver as early as 12 h after APAP treatment [22]. Interestingly, we found that PHH conditioned media following treatmentwith *H. hepaticus* for 24 h induced an increased expression of inflammatory cytokines (IL-8 and IL-1β) in monocytes isolated from peripheral blood (Fig. 6A-B). From this data we conclude that PHH are able to synthesize sufficient amounts of inflammatory cytokines in an early phase of inflammation to enable activation of non-resident monocytes. It is assumed that continuing inflammation in the liver leads to much higher concentrations of inflammatory mediators, produced to a substantial degree by specialized immune cells such as resident macrophages/Kupffer cells [23]. To rule out any contamination of isolated PHH by resident macrophages, we used an AlexaFluor488-labeled anti-human-CD68-antibody to stain for Kupffer cells in the isolated cell suspensions. As assessed by flow cytometry, no CD68-positive cells were present in freshly isolated PHH (data not shown). As a further control, in some experiments we carried out an additional purification step (percoll purification) as described elsewhere [24], in order to remove all contaminating non-parenchymal cells from the hepatocyte isolates. RT-PCR analysis revealed no significant difference of CD68 mRNA expression levels in freshly isolated PHH as compared to PHH isolated with the additional purification step (data not shown).

**Figure 7:**
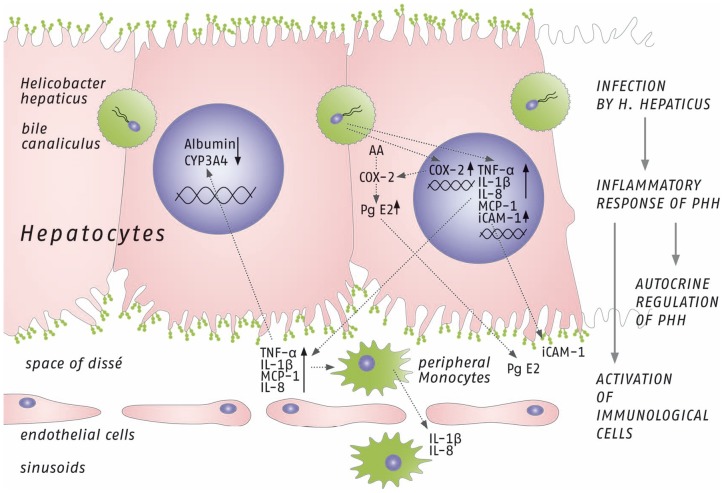
Schematic illustration showing the potential mechanism of the inflammatory response induced by *H. hepaticus* in PHH, summarizing main findings of this study. *In vivo*, infection of hepatocytes by *H. hepaticus* is thought to mainly occur via bile canaliculi, initiating an inflammatory response of PHH. First, gene expression of acute phase cytokines is induced (Monocyte chemotactic protein 1 (MCP-1), Interleukin 8 (IL-8), Tumor necrosis factor alpha (TNF-alpha) and Interleukin 1 beta (IL-1 beta)) and their perihepatic concentrations increase. Additionally, mRNA expression of cyclooxygenase 2 (COX-2) and intercellular adhesion molecule-1 (ICAM-1) are increased, resulting in higher perihepatic Prostaglandin E2 (PGE2) concentration and increased ICAM-1 incorporation into the cell membrane of hepatocytes. In a second step of the *in vivo* reaction sequence, peripheral monocytes are attracted and activated by newly synthetized acute phase cytokines, followed by induction of monocyte gene expression of Interleukin 1 beta (IL-1 beta) and Interleukin 8 (IL-8). Furthermore, acute phase cytokines most probably contribute to the downregulation of gene expression of albumin and cytochrome P450 3A4 of hepatocytes in an autocrine fashion. (AA  =  arachidonic acids).

Our study revealed an increased expression of COX-2 with a consecutively increased PGE2 concentration in the cell-culture supernatant after infection with *H. hepaticus* (Fig. 3A-B and 7). These inflammatory mediators might play an important role in activating and maintaining chronic inflammation in liver tissue which is associated with an increased risk of hepatic carcinogenesis [25]. The expression of COX-2 was found to be increased in human HCC [26]. Additionally, liver regeneration after partial hepatectomy promotes rapid expression of COX-2 and prostaglandin synthesis [27]–[28], suggesting a potential role of COX-2 and prostaglandins in linking injury-related inflammatory responses to subsequent protection and regeneration of liver tissue. Taking into account the large number of studies directly linking prostaglandins and inflammatory cytokines with various severe liver diseases, we believe that our observations concerning the initial phase of the inflammatory reaction of hepatocytes are of biological as well as of clinical relevance. It should be noted that the *in vitro* cell culture model for the *H. hepaticus* infection of PHH described here is somewhat limited by the short life span of *H. hepaticus* in this setting. *In vivo* studies would therefore be highly desirable for further investigations, e.g. investigating the impact of *H. hepaticus* on hepatic carcinogenesis.

Co-cultures of murine cells with *H. hepaticus* revealed strong pro-inflammatory responses in mouse liver cells and murine monocyte/macrophage cell lines, and to a lesser extent in mouse intestinal crypt cells [29]. Comparable to our data, that *H. hepaticus* initiates a very early inflammatory response in PHH, the release of the pro-inflammatory cytokine Macrophage inflammatory protein-2 into the cell culture supernatant of mouse liver cells was induced by whole *H. hepaticus* bacteria, *H. hepaticus* lysates as well as purified *H. hepaticus* LPS [29].

Although *Helicobacter spp*. have been identified in the liver of humans, their pathogenic role in human liver diseases remains still largely unclear [4]. Most case control trials in humans identified *Helicobacter spp*. only based on partial 16S ribosomal RNA gene sequence data or specific gene detection by PCR [4]. Unfortunately, this data does not provide conclusive evidence for *Helicobacter spp*. identification as recommended standards for describing *Helicobacter spp*. should additionally include phenotypic characteristics [30]. Successful isolation and cultivation of a *Helicobacter spp*. from a patient with liver cirrhosis was described in a case report [31]. Difficulties to culture *H. hepaticus* might be explained by the bacterial conversion from multiplying spiral to dormant but viable coccoid forms in a bile-rich environment [32]. In this context, it is remarkable that we were able to find apparently viable, motile *H. hepaticus* in co-cultivation with PHH in a suboptimal, non-microaerobic environment for up to 8 d, albeit with clearly declining overall motility and absolute number of vital bacteria (Videos S1-S3). Due to the difficulties in the cultivation of *H. hepaticus* from liver specimens, it would thus be desirable to use 2-PM in combination with Helicobacter-specific monoclonal antibodies for the identification of viable, i.e. highly motile, *Helicobacter spp*. within fresh intact liver tissue resection specimen of patients with different liver diseases.

Due to the suboptimal, non-microaerobic conditions necessary for the co-culture with PHH, the vitality of *H. hepaticus* obviously decreased, and consequently certain amounts of dead bacteria as well as bacterial fragments such as CpG-DNA of *H. hepaticus* presumably accumulated within the co-culture supernatants. In order to test if the observed PHH inflammatory response was predominantly activated by free DNA released from dying bacteria, we treated PHH with isolated DNA from *H. hepaticus* at a MOI of 20 bacterial DNA equivalents per cell. In this setting, no relevant increase in mRNA expression of inflammatory cytokines in PHH was found (data not shown).

The inflammatory response of *H. hepaticus*-infected PHH was largely subsiding by day 7, which is probably due to the largely reduced viability of *H. hepaticus* (see Video S3) because of unfavorable non-microaerobic culture conditions (see Methods). We assume that this decline in bacterial viability might in turn favor a recovery of the cultured PHH, thus at the same time preventing a progression towards chronic inflammation. Another factor that might have prevented the development of a full-fledged chronic inflammatory response is, of course, the absence of professional resident and migratory immune cells. Thus, apart from possible species-specific incompatibilities of *H. hepaticus* with the human organism, we conclude that our model of *in vitro*-infected PHH is well suited for studies focusing on the initial phase of human hepatocyte-specific acute inflammatory responses to bacterial infection in the liver.

As demonstrated by unchanged AST levels in the PHH culture supernatants, we believe that *H. hepaticus* does not induce necrosis or apoptosis of hepatocytes. Down-regulation of albumin synthesis as well as CYP3A4 expression in PHH following co-cultivation with *H. hepaticus* correlates with earlier experimental data of our group. We have shown previously that inflammatory cytokines (IL-6 and TNFα) decrease the expression of CYP enzymes and albumin in PHH [33]–[34]. Importantly, the impact of *H. hepaticus* on CYP3A4 expression is markedly stronger as compared to the treatment with recombinant human cytokines. The mRNA expression of CYP3A4 decreased to 27% compared with untreated hepatocytes after treatment with 10 ng/ml recombinant human IL-6 for 48 h [13]. In contrast, CYP3A4 gene expression in PHH decreased to as low as 3.3% after co-cultivation with *H. hepaticus* at a MOI of 100 bacteria per hepatocyte for 48 h (Fig. 5C). Garcia et al. showed in their mouse model of *H. hepaticus* liver infection that a decreased hepatic expression of P450 enzymes CYP2B10 and CYP3A11 was associated with an enhanced tumor promotion due to impaired metabolic detoxification of endobiotics, such as bile acids, and persistent microbial-induced immune response [35]. We believe that the observed CYP3A4 repression in our study is more likely a result of cytokine-dependent hepatocyte stimulation and not associated with tumor promotion. Increased cytokine concentrations in perihepatic space might influence the gene expression of CYP3A4 as well as of albumin by hepatocytes in an autocrine fashion (Fig. 7).

Based on the data of the novel *in vitro* culture model presented in this study, we conclude that *H. hepaticus* induces a very early inflammatory cytokine release (IL-1β, TNFα, IL-8 and MCP-1) in PHH. This is followed by the induction of COX-2 and an associated increase of PGE2 in the culture medium. Furthermore, the ICAM-1 surface expression on PHH is increased in response to the *H. hepaticus* infection. Changes in inflammatory gene expression and mRNA levels correlated well with the observed protein concentrations in cell-culture supernatants that were in turn able to activate isolated peripheral monocytes (Fig. 7).

Taken together, our findings indicate that *H. hepaticus* is able to induce an acute inflammatory reaction in the human liver and might contribute to the development of acute and/or chronic hepatitis, thus representing a potential risk factor for hepatic carcinogenesis.

Taken together, our findings demonstrate clearly a pathogenic effect of *Helicobacter spp*. on human liver tissue. Thus it can be assumed that the detection of *Helicobacter spp*. in human liver diseases was not in all cases just an independent coincidence or contamination from the upper gastrointestinal tract. The demonstrated strong inflammatory response of PHH co-cultured with *H. hepaticus* allows the assumption that *Helicobacter spp*. might contribute to the development of acute and/or chronic hepatitis, thus representing a potential risk factor for hepatic carcinogenesis. We therefore see the need for further studies on tissue from patients with chronic hepatitis, HCC or cholangiocellular carcinoma with the goal of precise identification of the *Helicobacter species* by cultivation.

## Material and Methods

### Ethics Statement

Written informed consent from each patient was obtained, and the study protocol was approved by the institutional ethical committee of Hannover Medical School.

### Hepatocyte isolation

Surgical liver supply, general anesthesia and isolation of human hepatocytes were carried out as previously described [33]–[34]. In brief, liver specimen of 8 donors obtained after partial hepatectomy were cannulated and flushed once with washing buffer containing 2.5 mM EGTA, followed by recirculating perfusion with digestion buffer containing 0.05% collagenase P (Roche). After mechanical tissue disruption, the resulting cell pellet (centrifuged at 50 g) was washed three times using PBS and resuspended in culture medium. Cell number and viability were determined by the Trypan blue exclusion test.

### Cell cultures and incubation

Primary human hepatocytes (PHH) with a viability of >80% were seeded in a rattail collagen sandwich in 6-well plates or 60 mm dishes at 1.3×10^6^ or 2.6×10^6^ viable cells/well, respectively. Supplemented William's medium E (1 µM insulin, 1 µM dexamethason/fortecortin, 100 U/ml penicillin, 100 µg/ml streptomycin, 1 mM sodium pyruvate, 15 mM HEPES buffer, 4 mM L-glutamine and 5% Fetal Calf Serum, all Biochrom) was used as culture medium. After 48 h, cells were washed, and medium without antibiotics was used for further cultivation. Five days after isolation, infection of PHH with *H. hepaticus* was performed using different multiplicities of infection (MOI). Incubation plates were centrifuged at 300 g for 3 min to mechanically deposit bacteria into the collagen layer, thus synchronizing infection. Supernatants were harvested after incubation periods of 2, 6, 24, 48 h and 7 d, respectively, and stored at −20°C until ELISA- and aspartate-aminotransferase (AST)-measurements. After incubation of adherent cell monolayers with collagenase type IV for 30 min, cells were scraped from the plates, washed twice with PBS, and stored at −80°C.

### Monocyte isolation and treatment

Human CD14^+^ monocytes were isolated from peripheral blood of 3 healthy donors according to manufacturer's instruction by positive selection using CD14-MACS-MicroBeads (Miltenyi). Cells were washed once and subsequently seeded at 10.000 cells/well in 96-well plates. Monocytes were stimulated 48 h with 100 µl of centrifuged PHH conditioned media following infection with *H. hepaticus* for 48 h. Adherent monocytes were scraped from the plates and after washing twice in PBS stored at −80°C.

### Bacteria and growth conditions

*H. hepaticus* strain ATCC 51449 (“strain 3B1”, as sequenced by Suerbaum et al. 2003) was used for infection of PHH cultures (36). *H. hepaticus* was cultured under specific microaerobic conditions (10% CO_2_, 80% N_2_, 10% H_2_) on blood agar plates (Columbia agar base II; Oxoid) supplemented with 10% horse blood and the following antibiotics: vancomycin (10 mg/l), polymyxin B (2500 U/l), trimethoprim (5 mg/l), amphotericin B (4 mg/l), as previously described [37]. Unless indicated otherwise, bacteria were preincubated on plates for 24 h at 37°C under microaerobic conditions for infection assays.

### Two-photon-microscopy (2-PM)

At various times of co-cultivation, time-lapse series were aquired by 2-PM using a TriM Scope-based upright microscope (LaVision/Olympus) fitted with a 20× water immersion objective, 485 and 600 nm long pass filters, and a MaiTai HP Ti:Sa laser (Spectra-Physics) tuned to 760 or 800 nm. Imaging volume was 75×75 µm or 150×150 µm (XY),12–40 µm, 2 to 4 slices (Z). Acquisition speed was 1 frame/3–6 sec, 4D image data analysis was done in Imaris (Bitplane).

### Real-time reverse transcriptase polymerase chain reaction (Real-Time PCR)

RNA was isolated using the NucleoSpin RNA2-Kit (Machery-Nagel) according to manufacturer's recommendations. 4 µg of total RNA were used for reverse transcription with the Omniscript kit (Qiagen). Real-Time PCR was performed on a StepOne Plus real-time PCR system (Applied Biosystems). To determine relative expression levels of target genes, Ct values were normalized against house-keeping genes (β-actin for PHH and Hypoxanthine-Phosphoribosyl-Transferase-1 for monocytes), and compared to control cultures using the δδCt value to calculate relative expression [38]. The following primers/probes (Applied Biosystems) were used: β-actin: Hs99999903_m1; Cytochrome P450 (CYP) 3A4: Hs00604506_m1; MCP-1: Hs00234140_m1; TNFα: Hs00174128_m1; IL-8: Hs00174103_m1; IL-1β: Hs01555410_m1; COX-2: Hs00153133_m1; albumin: Hs00910225_m1; ICAM-1: Hs00164932_m1; Hypoxanthine-Phosphoribosyl-Transferase-1: Hs02800695_m1.

### Enzyme linked immunosorbent assay (ELISA)

PHH supernatants were analysed for human albumin using an ELISA kit (Bethyl Laboratories), and for Prostaglandin E2 (PGE 2) using a PGE2 direct Biotrak assay (GE Healthcare). Protein concentrations of cytokines released by PHH were measured by human IL-8 and MCP-1 ELISA kits (both eBioscience). All measurements were performed according to the manufacturer's instructions.

### Flow cytometry

PHH were harvested by incubation with collagenase type IV as described above. After PBS washing, cells were suspendend at 20.000 cells/100 µl and incubated for 2 h with AlexaFluor488-labeled anti-human-ICAM-1-antibody (Biolegend) diluted to 1∶50. Cell surface staining was analyzed using a FACSCalibur (BD).

### AST detection in the supernatant of PHH

As an indicator of cell damage, AST activities were measured in PHH supernatants by standardized enzyme activity assays (Roche Molecular Diagnostics) performed by the central laboratory of Hannover Medical School.

### Statistical analysis

All Real-Time PCRs were reproducible and carried out in duplicate. Each set of experiments was repeated at least three times with hepatocytes from different donors. Results are presented as means, significance values were calculated by one-way ANOVA. A p-value of <0.05 was considered significant.

## Supporting Information

Video S1 **Two-photon-microscopy of** ***H. hepaticus*** **ATCC 51449 co-cultivated with primary human hepatocytes (PHH) for 6 hours.** PHH were co-cultured with *H. hepaticus* at a MOI of 100 per hepatocyte for 6 h. Cell nuclei and *H. hepaticus* were stained with DAPI (white). Starting positions of individual bacteria are marked with red dots, tracks of bacterial movement are displayed as red lines. At 6 hours of co-cultivation, numerous bacteria are present in the vicinity of hepatocytes, displaying a highly active, seemingly random motility. 2-PM settings: TiSa laser at 800 nm, 485 and 600 nm long pass filters, imaging volume 150×150 µm (XY), 12–40 µm, 2–4 Z-slices (Z), acquisition speed 1 frame/3–6 sec, 40× replay speed.
(MOV)

Video S2 **Two-photon-microscopy of** ***H. hepaticus*** **ATCC 51449 co-cultivated with primary human hepatocytes (PHH) for 3 days.** PHH were co-cultured with *H. hepaticus* at a MOI of 100 per hepatocyte for 3d. ICAM-1 on hepatocyte cell membranes was stained using an AlexaFluor488-labeled anti-human-ICAM-1-antibody (green). Cell nuclei and *H. hepaticus* were stained with DAPI (white). Starting positions of individual bacteria are marked with red dots, tracks of bacterial movement are displayed as red lines. Following 3 days of co-cultivation, high levels of ICAM-1 are expressed on hepatocyte cell membranes, while most bacteria present in the culture surrounding PHH continue to exihibit a high overall motility. 2-PM settings: TiSa laser at 800 nm, 485 and 600 nm long pass filters, imaging volume 150×150 µm (XY), 12–40 µm, 2–4 Z-slices (Z), acquisition speed 1 frame/3–6 sec, 40× replay speed.
(MOV)

Video S3 **Two-photon-microscopy of H. hepaticus ATCC 51449 co-cultivated with primary human hepatocytes (PHH) for 8 days.** PHH were co-cultured with H. hepaticus at a MOI of 100 per hepatocyte for 8 d. ICAM-1 on hepatocyte cell membranes was stained using an AlexaFluor488-labeled anti-human-ICAM-1-antibody (green). Cell nuclei and H. hepaticus were stained with DAPI (white). Starting positions of individual bacteria are marked with red dots, tracks of bacterial movement are displayed as red lines. After 8 days of infection, ICAM-1-staining on hepatocytes is comparable to day 3, while the remaining bacteria were observed to be largely non-motile. 2-PM settings: TiSa laser at 800 nm, 485 and 600 nm long pass filters, imaging volume 150×150 µm (XY), 12–40 µm, 2–4 Z-slices (Z), acquisition speed 1 frame/3–6 sec, 40× replay speed.
(MOV)
